# West Highland White Terriers under primary veterinary care in the UK in 2016: demography, mortality and disorders

**DOI:** 10.1186/s40575-019-0075-2

**Published:** 2019-09-03

**Authors:** Dan G. O’Neill, Zoie F. Ballantyne, Anke Hendricks, David B. Church, Dave C. Brodbelt, Camilla Pegram

**Affiliations:** 1 Pathobiology and Population Science, The Royal Veterinary College, Hawkshead Lane, North Mymms, Hatfield, Herts AL9 7TA UK; 2 Clinical Sciences and Services, The Royal Veterinary College, Hawkshead Lane, North Mymms, Hatfield, Herts AL9 7TA UK

**Keywords:** VetCompass, Electronic patient record, EPR, Breed, Dog, Epidemiology, Primary-care, Veterinary, Pedigree, Purebred, Atopy, Dermatology, Skin

## Abstract

**Background:**

The West Highland White Terrier (WHWT) is a relatively common breed in the UK, although Kennel Club registrations have declined in recent years. The VetCompass™ Programme collates de-identified clinical data from primary-care veterinary practices in the UK for epidemiological research. Using VetCompass clinical data, this study aimed to characterise the demography, longevity and common disorders of WHWTs under primary veterinary care in the UK.

**Results:**

WHWTs comprised 6605/905,544 (0.7%) dogs under veterinary care during 2016 from 886 clinics. Mean adult bodyweight was 9.6 kg (standard deviation [SD] 1.8 kg). Males (10.1 kg, SD 1.8 kg) were heavier than females (9.0 kg, SD 1.6 kg) (*P* < 0.001). Median age was 7.8 years (interquartile range [IQR] 4.3–11.1). Median longevity was 13.4 years (IQR 11.0–15.0). Males (13.8 years) outlived females (12.9 years) (*P* = 0.045). The most common grouped causes of death were lower respiratory tract (10.2, 95% CI: 5.5–16.7), neoplastic (10.2, 95% CI: 5.5–16.7) and spinal cord disorder (7.8, 95% CI: 3.8–13.9). Overall, 71.5% WHWTs had > 1 disorder recorded during 2016. The most prevalent specific disorders were periodontal disease (15.7, 95% CI: 14.1–17.3), otitis externa (10.6, 95% CI: 9.3–12.0), overgrown nails (7.2, 95% CI: 6.2–8.4), allergic skin disorder (6.5, 95% CI: 5.5–7.7) and obesity (6.1, 95% CI: 5.1–7.2). The most prevalent grouped disorders were cutaneous (22.7, 95% CI: 20.9–24.6), dental (17.8, 95% CI: 16.2–19.6) and aural (12.3, 95% CI: 11.0–13.8). The median age of dogs affected with the 27 most common disorders varied from 6.7 (pododermatitis) to 13.9 years for cataracts.

**Conclusions:**

These findings highlight that, despite a recent decline in popularity, WHWTs are still relatively common in the UK. Dental disease, ear disease, overgrown nails, allergic skin disorder and obesity were identified as common health issues within the breed. Cutaneous disorders were the most common disorder group in the breed but showed a lower prevalence than might be expected. These results can be used by breeders, veterinary practitioners and owners as an evidence base to predict, prevent and manage key health and welfare issues for WHWTs.

## Plain English Summary

West Highland White Terriers (WHWTs) are a relatively common dog breed in the UK, although Kennel Club registrations have declined in recent years. There are reported predispositions in WHWTs to 42 disorders, most notably skin disorders. However predisposition does not necessarily mean that a disease is common or even important for the breed. Using veterinary clinical data from the VetCompass™ Programme at the Royal Veterinary College, this study aimed to describe the demography and frequency of common disorders of WHWTs under primary veterinary care in the UK.

WHWTs comprised 6605 (0.7%) of the overall 905,544 study dogs. Males (10.1 kg) were heavier than females (9.0 kg). The average lifespan was 13.4 years, with males (13.8 years) outliving females (12.9 years). The most common causes of death were lower respiratory tract disorder (10.2%), cancer (10.2%) and spinal cord disorder (7.8%). Overall, 71.5% WHWTs had more than one disorder recorded during 2016. The most common disorders were dental disease (15.7%), ear disease (10.6%), overgrown nails (7.2%), allergic skin disorder (6.5%) and overweight (6.1%). Skin disorders were most common disorder grouped (22.7%).

This study documents the WHWT as still being a relatively common dog breed in the UK, although its popularity is in steep decline. The WHWT is shown to be a long-lived breed. Dental disease, ear disease, overgrown nails, allergic skin disorder and overweight were identified as common health issues within the breed. Skin disorders were the most common health group issue. These results can provide useful evidence to veterinarians and owners in order to improve the health and welfare of WHWTs.

## Background

The West Highland White Terrier (WHWT) was developed from a white strain of Cairn Terrier bloodlines in the mid-19th century in Argyllshire, Scotland. Over time, the breed gradually moved towards a shorter body and higher tail carriage than the traditional Cairn Terrier and was recognized formally as a distinct breed in 1907 by the Kennel Club (KC) [1]. Although still a relatively common breed in the UK, KC registrations for WHWTs have declined in recent years. During the 10-year period 2008–2017 inclusive, registrations decreased 70.8% from 2.7 to 0.9% of total KC registrations. In contrast, the same 10-year period oversaw substantial increases in registrations for some other similar-sized breeds to the WHWT, including the Pug, Bulldog, French Bulldog and Miniature Smooth Dachshund suggesting competing impacts from appeal for the same group of potential owners [2]. However, these data just describe the KC registered subset of the breed and do not take account of changing popularity among the wider, and often non-registered, subset of the WHWT population that have been largely unaccounted to date.

The WHWT is considered a long-lived breed with a median longevity of 13.5 years that compares favourably with the median of 12.0 across all breeds [3]. A survey of owners of pedigree dogs in the UK identified the most common causes of death/reasons for euthanasia in WHWTs as old age (14.6%) and kidney failure (8.3%) [4]. This contrasts with an owner survey of the general population of WHWTs in the US that reported the most common diseases WHWTs died with as atopic dermatitis (31.1%), pulmonary fibrosis (10.5%) and congestive heart failure (9.1%) [5]. Despite impressive longevity, the WHWT is reportly predisposed to 42 disorders including aggression, atopic dermatitis, craniomandibular osteopathy, keratoconjunctivitis sicca and bronchiectasis [6]. The WHWT is reported as the dog breed with the second-lowest level of genetic heterozygosity in the UK suggesting a high level of inbreeding which may be associated with some of these disease predispositions, although there is not a clear correlation between heterozygosity and the level and severity of inherited disease overall within breeds [7, 8]. In a WHWT health survey in the US, owners reported atopic dermatitis as the most common disease of this breed (23.2% affected) followed by luxated patella (5.5%) and aggression (3.9%) [5]. These results differ slightly to a KC breed health survey in the UK where the most commonly reported conditions were hypersensitivity (allergic) skin disorder (8.6%), dermatitis (4.7%), chronic itching (3.8%) and otitis externa (2.9%), although it is possible that many of these reported conditions were related to underlying allergic dermatitis and otitis [4]. Keratoconjunctivitis sicca (2.4%), cruciate disease (1.8%) and patellar luxation (1.8%) were other commonly recorded disorders in the UK [4].

The KC’s Breed Watch scheme serves as an ‘early warning system’ to identify points of concern for individual breeds and classifies WHWT as a category 2 breed with two points of concern for special attention by judges: skin irritation and misplaced lower canines [9]. The WHWT has been identified world-wide as a breed predisposed to canine atopic dermatitis [10] albeit with some variability in the level of predisposition between populations in different geographic locations [10, 11]. Atopic dermatitis is a chronic, inflammatory and pruritic skin disease with a complex pathogenesis that includes allergic pathways in the majority of affected dogs [12]. It is recognised that the life-long nature of the disease, its complications, and associated financial costs are challenging, even a burden, for affected dogs and their carers, and a challenge for clinicians [13, 14], despite the expanding treatment options for this disease [15]. A recent study that developed a metric to summarise welfare compromise and total impact of disease in individual animals estimated that atopic dermatitis had by far the highest score out of the 10 canine diseases studied, even though euthanasia due to the disease was not factored into the estimate [16]. The documented breed predisposition and impact of this kind of allergic skin disease is reflected in the focus on skin disease by the KC as a priority for the WHWT, although much of the current evidence has been derived from referral veterinary care and owner survey data sources [9]. Clinical data derived from primary veterinary care databases has been suggested to offer an additional perspective that combines the strengths of veterinary-quality diagnoses with study populations that are representative of the wider population [17, 18].

Using veterinary clinical data from the VetCompass™ Programme [19], this study aimed to characterise the demography, longevity and common disorders of West Highland White Terriers under primary veterinary care in the UK. The study placed special focus on exploration of health associations with age and sex. These results could assist breeders, veterinary practitioners and owners with an evidence base on the wider general population to predict, prevent and manage key health and welfare opportunities for WHWTs.

## Materials and methods

The study population included all available dogs under primary veterinary care at clinics participating in the VetCompass Programme during 2016. Dogs under veterinary care were defined as those with either a) at least one electronic patient record (EPR) (free-text clinical note, treatment or bodyweight) recorded during 2016 or b) at least one EPR recorded during both 2015 and 2017. VetCompass collates de-identified electronic patient record (EPR) data from primary-care veterinary practices in the UK for epidemiological research [19]. Data fields available for the study included a unique animal identifier along with species, breed, date of birth, sex, neuter status and bodyweight, and also clinical information from free-form text clinical notes, summary diagnosis terms [20] and treatment with relevant dates.

A cohort study design was used to estimate the one-year (2016) period prevalence of the most commonly diagnosed disorders [21]. Sample size calculations estimated that 2069 dogs would need to be sampled from a population of 6605 dogs in order to estimate the prevalence of a disorder affecting 3% of the dogs with 0.5% acceptable margin of error at a 95% confidence level [22]. Ethics approval was obtained from the RVC Ethics and Welfare Committee (reference number 2015/1369).

Dogs recorded as West Highland White Terrier breed were categorised as West Highland White Terrier and all remaining dogs were categorised as non-West Highland White Terrier. No distinction was made between Kennel Club registered and unregistered individuals. *Adult Bodyweight* described the mean bodyweight (Kg) recorded from all bodyweight data for dogs aged over 18 months at the time of weighing and was categorised into 5 groups (< 7.0, 7.0 to < 9.0, 9.0 to < 11.0, 11.0 to < 13.0, ≥ 13.0). N*euter* described the status of the dog (entire or neutered) at the final EPR. *Age* described the age (years) at the final date under veterinary care during 2016 (December 31st, 2016) and was categorised into 5 groups (< 3.0, 3.0 to < 6.0, 6.0 to < 9.0, 9.0 to < 12.0, ≥ 12.0).

The list of unique West Highland White Terrier animal identification numbers was randomly ordered and the clinical records of a randomly selected sample of animals were reviewed manually in detail to extract the most definitive diagnoses for all disorders recorded as existing during 2016 as previously described [23]. Elective (e.g. neutering) or prophylactic (e.g. vaccination) clinical events were not included. No distinction was made between pre-existing and incident disorder presentations. Disorders described within the clinical notes using presenting sign terms (e.g. ‘vomiting’ or ‘vomiting and diarrhoea’), but without a formally recorded biomedical diagnostic term, were included using the first sign listed (e.g. vomiting). Mortality data (recorded cause, date and method of death) were extracted on all deaths at any date during the available EPR data. The extracted diagnosis terms were mapped to a dual hierarchy of diagnostic precision for analysis: fine-level and grouped-level as previously described [23]. Briefly, fine-level terms described the original extracted terms at the maximal diagnostic precision recorded within the clinical notes (e.g. *inflammatory bowel disease* would remain as *inflammatory bowel disease*). Grouped-level terms mapped the original diagnosis terms to a general level of diagnostic precision (e.g. *inflammatory bowel disease* would map to *gastro-intestinal*).

Following data checking for internal validity and cleaning in Excel (Microsoft Office Excel 2013, Microsoft Corp.), analyses were conducted using Stata Version 13 (Stata Corporation). The sex, neuter status, age and adult bodyweight for WHWTs under veterinary care during 2016 were described. Annual proportional birth rates described the relative proportion of WHWTs compared with all dogs that were born in each year from 2004 to 2015 from the cohort of dogs under veterinary care in 2016. All-age bodyweight data with associated dates were used to generate individual bodyweight growth curves for male and female WHWTs by plotting age-specific bodyweights and were overlaid with a cross medians line plot using the Stata *mband* command.

One-year period prevalence values were reported along with 95% confidence intervals (CI) that described the probability of diagnosis at least once during 2016. The CI estimates were derived from standard errors based on approximation to the binomial distribution [24]. The median age at the end of the study date range was reported for affected animals. Prevalence values were reported overall and also separately for males and females. The chi-square test was used to compare categorical variables and the Students t-test or Mann-Whitney U test to compare continuous variables as appropriate [24]. Statistical significance was set at the 5% level. The mean was reported for continuous data that were normally distributed; otherwise the median was reported [24].

## Results

### Demography and mortality

**Fig. 1:**
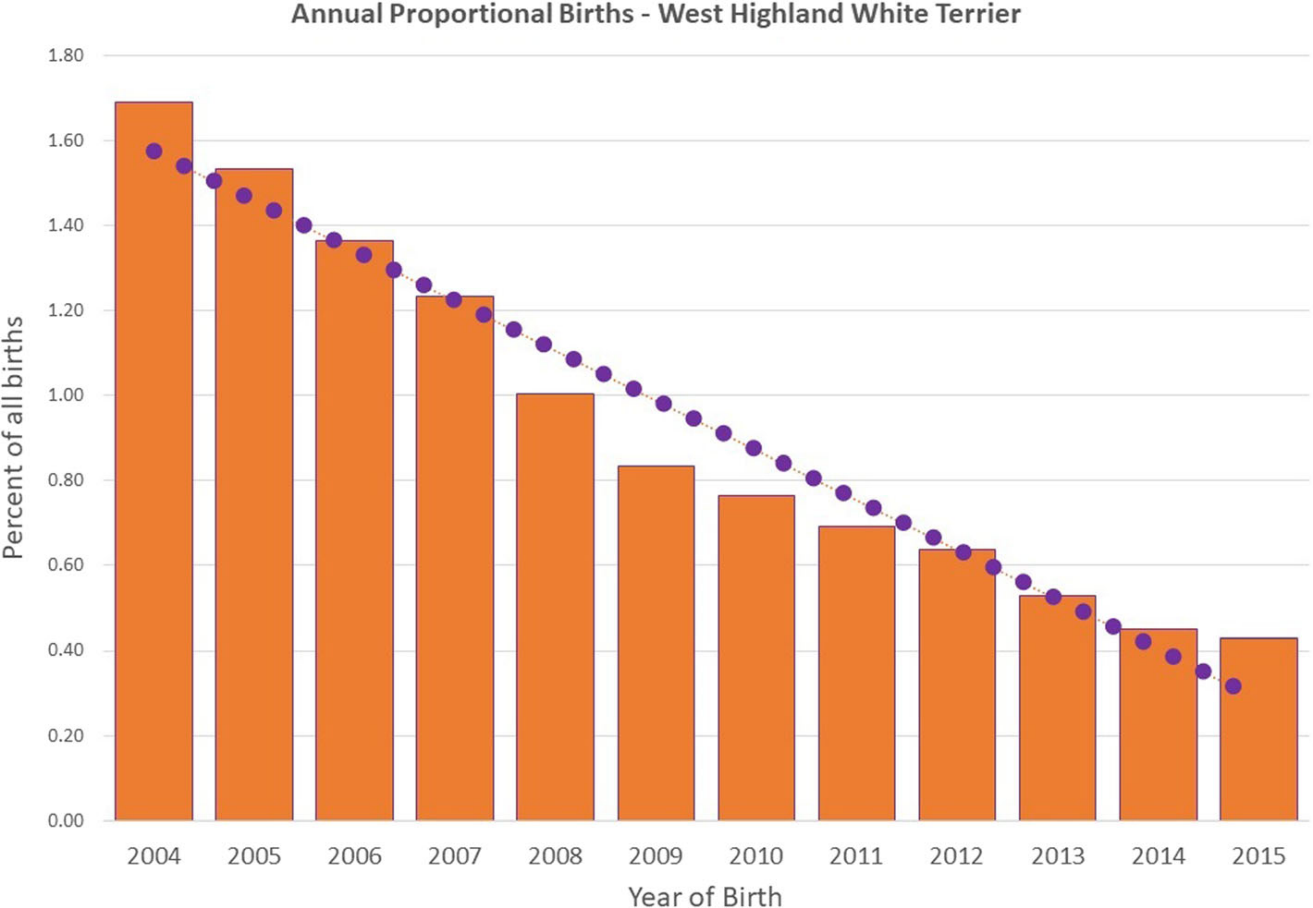
Annual proportional birth rates (2004–2015) for West Highland White Terriers (*n* = 6605) among all dogs (*n* = 905,544) under UK primary veterinary care from January 1st 2016 to December 31st, 2016 at practices participating in the VetCompass™ Programme

**Fig. 2:**
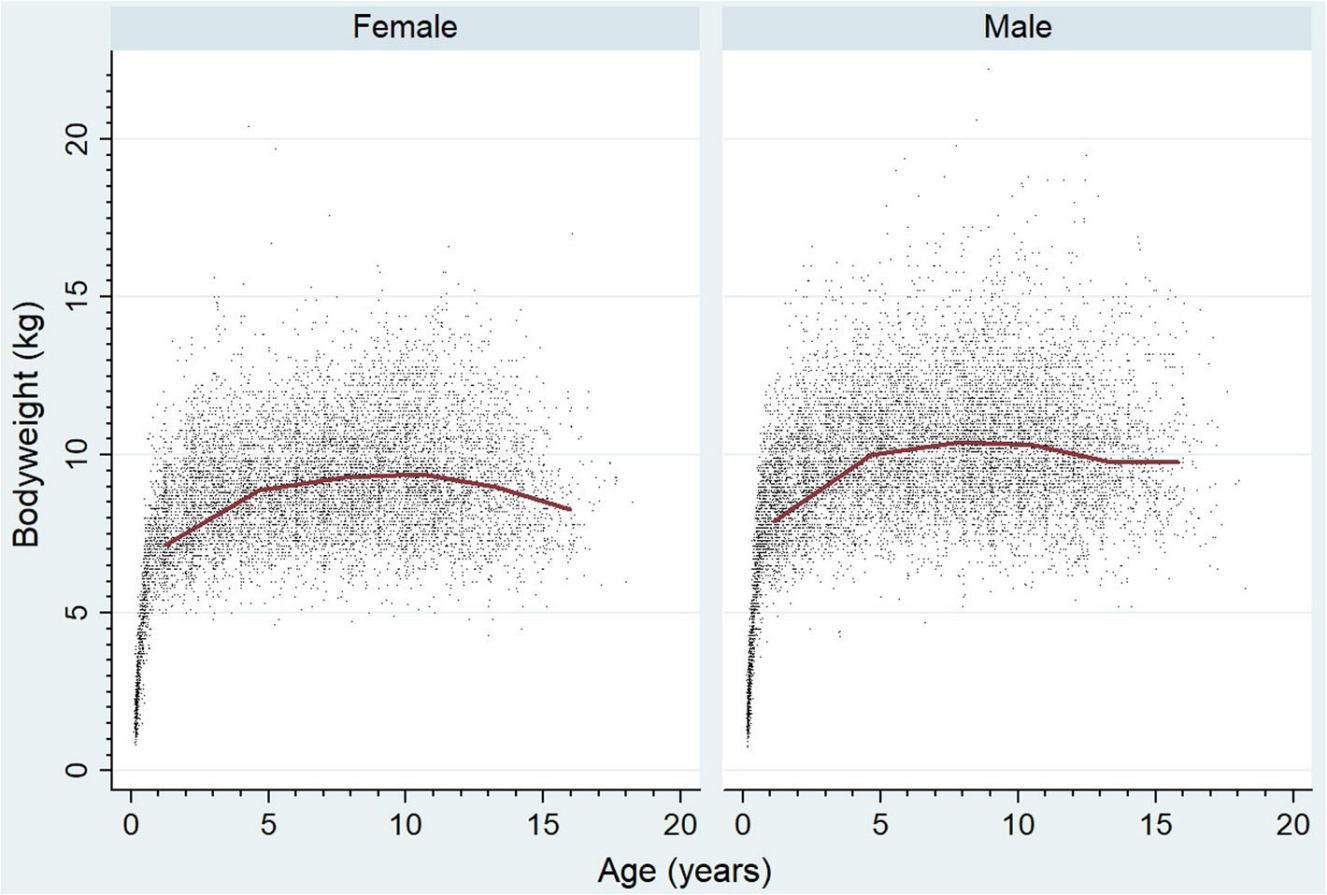
Bodyweight growth curves overlaid with a cross medians line plot for female and male West Highland White Terriers under UK primary veterinary care from January 1st 2016 to December 31st, 2016 at practices participating in the VetCompass™ Programme (10,416 bodyweight values from 2451 females and 11,907 bodyweight values from 2822 males)

The study population of 905,544 dogs under veterinary care attending 886 clinics in the vetCompass database during 2016 included 6605 (0.7%) WHWTs. Annual proportional birth rates showed that WHWTs decreased in popularity from 1.69% of the annual VetCompass birth cohort in 2004 to 0.43% in 2015 (Fig. 1). Of these 6605 WHWTs with information recorded, 3090 (46.9%) were female and there were 3455 (52.4%) animals across both sexes neutered. The proportion neutered did not differ between females (53.4%) and males (51.6%) (*P* = 0.132). The median age of the WHWTs overall was 7.8 years (interquartile range [IQR] 4.3–11.1, range 0.4–20.7). The median age of females (7.8 years) did not differ to males (7.8 years) (*P* = 0.365). The mean adult bodyweight overall was 9.6 kg (standard deviation [SD] 1.8 kg). The mean adult bodyweight of males (10.1 kg, SD 1.8 kg) was heavier than females (9.0 kg, SD 1.6 kg) (*P* < 0.001) (Table 1). The median bodyweight across all ages for males (9.6 kg, IQR: 8.2–11.0, range: 0.8–22.2) was higher than for females (8.8 kg, IQR: 7.5–10.0, range: 0.8–20.4) (*P* < 0.001). Bodyweight growth curves based on 10,416 bodyweight values from 2451 females and 11,907 bodyweight values from 2822 males showed that WHWT puppies grow rapidly up to 1 year of age but continue to gain further weight up to 5 years of age before leveling off thereafter (Fig. 2). Data completeness varied across the variables assessed: age 98.2%, sex 99.7%, neuter 99.7% and bodyweight at any age 80.0%.

**Table 1:** Demography of 6605 West Highland White Terriers under UK primary veterinary care from January 1st 2016 to December 31st, 2016 at practices participating in the VetCompass™ Programme

Variable	Category	Count^a^	Percent
Sex	Female	3090	46.9
	Male	3498	53.1
Female neuter	Entire	1439	46.6
	Neutered	1651	53.4
Male neuter	Entire	1694	48.4
	Neutered	1804	51.6
Female adult bodyweight (aged ≥18 months) (kg)	< 7.0	195	8.5
	7.0 to < 9.0	988	42.9
	9.0 to < 11.0	829	36.0
	11.0 to < 13.0	249	10.8
	≥ 13.0	40	1.7
Male adult bodyweight (aged ≥18 months) (kg)	< 7.0	54	2.1
	7.0 to < 9.0	659	25.4
	9.0 to < 11.0	1.152	44.4
	11.0 to < 13.0	561	21.6
	≥ 13.0	169	6.5
Age (years)	< 3.0	1055	16.3
	3.0 to < 6.0	1367	21.1
	6.0 to < 9.0	1393	21.5
	9.0 to < 12.0	1480	22.8
	≥ 12.0	1194	18.4

^a^Count covers dogs with available data

There were 164 deaths recorded during the study. The median longevity overall was 13.4 years (IQR 11.0–15.0, range 3.2–19.6). The median longevity of males (*n* = 96, 13.8 years, IQR 11.1–15.3, range 4.7–18.6,) was greater than females (*n* = 64, 12.9 years, IQR 10.3–14.7, range 3.2–19.6) (*P* = 0.045). The method of death was recorded in 159 (97.0%) of deaths. Of these, euthanasia accounted for 148 (93.1%) deaths while 11 (6.9%) were unassisted. Of the 128 (88.0%) deaths with a cause recorded, the most common causes of death described at a grouped-level were lower respiratory tract (*n* = 13, prevalence 10.2%), neoplastic (13, 10.2%) and spinal cord disorders (10, 7.8%) (Table 2).

**Table 2:** Mortality in West Highland White Terriers with a recorded cause of death under UK primary veterinary care at practices participating in the VetCompass™ Programme from January 1st 2016 to December 31st, 2016 (*n* = 128)

Grouped-level disorder	Count	Percent	95% CI
Lower respiratory tract	13	10.2	5.5–16.7
Neoplastic	13	10.2	5.5–16.7
Spinal cord	10	7.8	3.8–13.9
Behavioural	8	6.3	2.7–11.9
Brain	8	6.3	2.7–11.9
Enteropathy	8	6.3	2.7–11.9
Appetite-related	7	5.5	2.2–10.9
Collapsed	7	5.5	2.2–10.9
Cardiac	7	5.5	2.2–10.9
Urinary system	7	5.5	2.2–10.9
Renal	6	4.7	1.7–9.9
Mass associated	5	3.9	1.3–8.9
Aural	4	3.1	0.9–7.8
Cutaneous	4	3.1	0.9–7.8
Others	21	16.4	10.5–24.0

### Disorder prevalence

The EPRs of a random sample of 2058/6605 (31.2%) WHWTs were manually examined to extract all recorded disorders for 2016. There were 1471 (71.5%) WHWTs with at least one disorder recorded during 2016. The remaining 28.5% had no disorder recorded and either presented for prophylactic management only or did not present at all during 2016. The median annual disorder count per WHWT during 2016 was 1 disorder (IQR 0–2, range 0–10). The median annual disorder count did not vary between females (1, IQR 0–2, range 0–10) and males (1, IQR 0–2, range 0–8) (*P* = 0.986).

The study included 3006 unique disorder events recorded during 2016 that encompassed 293 distinct fine-level disorder terms. The most prevalent fine-level disorders recorded were periodontal disease (*n* = 323, prevalence 15.7, 95% CI: 14.1–17.3), otitis externa (218, 10.6, 95% CI: 9.3–12.0), overgrown nails (149, 7.2, 95% CI: 6.2–8.4), allergic skin disorder (134, 6.5, 95% CI 5.5–7.7) and obesity (126, 6.1, 95% CI 5.1–7.2). Among the 27 most common fine-level disorders, males had higher probability than females for two disorders (otitis externa, and aggression) while females had higher prevalence for one (periodontal disease). The median age of dogs affected with specific fine-level disorders among the 27 most common disorders varied from 6.7 years for pododermatitis to 13.9 years for cataracts (Table 3).

**Table 3:** Prevalence of the most common disorders at a *fine-level of diagnostic precision* recorded in West Highland White Terriers (*n* = 2058) under UK primary veterinary care from January 1st 2016 to December 31st, 2016 at practices participating in the VetCompass™ Programme. The *P*-value reflects prevalence comparison between females and males

Fine-level disorder	Count	Overall prevalence %	95% CI^a^	Female prevalence %	Male prevalence %	*P*-Value^b^	Median age (years)
Periodontal disease	323	15.7	14.1–17.3	17.7	14.0	**0.024**	9.4
Otitis externa	218	10.6	9.3–12.0	8.7	12.2	**0.011**	8.7
Overgrown nails	149	7.2	6.2–8.4	8.2	6.5	0.144	8.4
Allergic skin disorder	134	6.5	5.5–7.7	5.9	7.0	0.319	8.1
Obesity	126	6.1	5.1–7.2	7.1	5.3	0.092	7.2
Anal sac impaction	105	5.1	4.2–6.1	5.9	4.4	0.118	7.3
Pododermatitis	88	4.3	3.4–5.2	3.8	4.7	0.333	6.7
Flea infestation	62	3.0	2.3–3.8	3.0	3.1	0.901	7.3
Pyoderma	55	2.7	2.0–3.5	2.0	3.2	0.086	9.8
Osteoarthritis	53	2.6	1.9–3.6	2.7	2.5	0.855	11.5
Atopic dermatitis	51	2.5	1.9–3.2	2.0	2.9	0.208	8.7
Diarrhoea	50	2.4	1.8–3.2	2.5	2.3	0.767	8.2
Aggression	41	2.0	1.4–2.7	1.1	2.8	**0.005**	7.8
Keratoconjunctivitis sicca	41	2.0	1.4–2.7	2.4	1.6	0.187	10.8
Dental disease	40	1.9	1.4–2.6	1.8	2.1	0.660	7.4
Haircoat disorder	40	1.9	1.4–2.6	2.4	1.5	0.138	7.6
Cruciate disease	38	1.8	1.3–2.5	2.3	1.4	0.135	8.4
Dermatitis	37	1.8	1.3–2.5	1.4	2.2	0.183	8.6
Vomiting	36	1.7	1.2–2.4	2.3	1.3	0.065	9.2
Conjunctivitis	28	1.4	0.9–2.0	1.5	1.3	0.664	7.4
Aural discharge	28	1.4	0.9–2.0	1.5	1.3	0.664	7.7
Skin mass	27	1.3	0.9–1.9	1.1	1.5	0.350	10.7
Ticks infestation	26	1.3	0.8–1.8	1.5	1.1	0.415	7.8
Cataract	25	1.2	0.8–1.8	1.6	0.9	0.156	13.9
Diabetes mellitus	25	1.2	0.8–1.8	1.3	1.2	0.835	11.2
Gastroenteritis	24	1.2	0.7–1.7	1.4	1.0	0.416	7.5
Skin cyst	24	1.2	0.7–1.7	0.9	1.4	0.213	10.1

^a^CI confidence interval ^b^*P*-Values in bold highlight disorders that differ in prevalence between males and females

There were 52 distinct grouped-level disorder terms recorded. The most prevalent grouped-level disorders were cutaneous (*n* = 468, prevalence: 22.7, 95% CI: 20.9–24.6), dental (367, 17.8, 95% CI: 16.2–19.6), aural (254, 12.3, 95% CI: 11.0–13.8), claw/nail (179, 8.7, 95% CI 7.5–10.0) and musculoskeletal (162, 7.9, 95% CI: 6.7–9.1) disorders. Among the 20 most common grouped disorders, males had higher probability than females for two disorders (cutaneous and aural disorders) while females had higher prevalence also for two disorders (lower respiratory tract and urinary disorders) The median age of dogs affected with specific grouped-level disorders varied from 5.3 years for traumatic to 12.7 years for urinary disorders (Table 4).

**Table 4:** Prevalence of the most common disorders at a *grouped-level of diagnostic precision* recorded in West Highland White Terriers (*n* = 2058) under UK primary veterinary care from January 1st 2016 to December 31st, 2016 at practices participating in the VetCompass™ Programme. The *P*-value reflects prevalence comparison between females and males

Grouped-level disorder	Count	Overall prevalence %	95% CI^a^	Female prevalence %	Male prevalence %	*P*-Value^b^	Median age (years)
Cutaneous	468	22.7	20.9–24.6	19.9	25.2	**0.004**	8.3
Dental	367	17.8	16.2–19.6	19.4	16.6	0.096	8.9
Aural	254	12.3	11.0–13.8	10.5	13.9	**0.020**	8.9
Claw/nail	179	8.7	7.5–10.0	9.3	8.2	0.365	8.4
Musculoskeletal	162	7.9	6.7–9.1	8.9	7.0	0.115	9.3
Enteropathic	146	7.1	6.0–8.3	8.2	6.2	0.087	8.0
Ophthalmological	143	6.9	5.9–8.1	7.5	6.5	0.356	10.8
Anal sac	127	6.2	5.2–7.3	6.9	5.6	0.221	7.3
Obesity	126	6.1	5.1–7.2	7.1	5.3	0.092	7.2
Mass-associated	109	5.3	4.4–6.4	5.9	4.7	0.205	11.7
Neoplastic	101	4.9	4.0–5.9	4.5	5.2	0.418	11.3
Parasite infestation	96	4.7	3.8–5.7	5.0	4.4	0.543	7.3
Behavioural	77	3.7	3.0–4.7	2.9	4.5	0.051	7.6
Lower respiratory tract	45	2.2	1.6–2.9	3.2	1.4	**0.005**	11.8
Cardiac	40	1.9	1.4–2.6	1.9	2.0	0.904	12.5
Upper respiratory tract	39	1.9	1.4–2.6	1.4	2.3	0.111	8.9
Endocrine	38	1.8	1.3–2.5	2.1	1.6	0.403	11.1
Urinary	38	1.8	1.3–2.5	2.9	1.0	**0.002**	12.7
Traumatic	33	1.6	1.1–2.2	1.2	2.0	0.143	5.3

^a^CI confidence interval ^b^*P*-Values in bold highlight disorders that differ in prevalence between males and females

## Discussion

This is the largest study to date using primary-care veterinary data to report on WHWT health. The study characterised the demography of 6605 dogs and the longevity and common disorders of 2058 WHWTs under primary veterinary care in the UK. The median age of WHWTs was 7.8 years, suggesting that this was an ageing population compared with previously reported median ages of Labrador Retrievers (4.9 years) [25], German Shepherd Dogs (4.7 years) [26] and Rottweilers (4.5 years) [27] which showed younger profiles. The most common causes of mortality were lower respiratory tract disease, neoplasia and spinal cord disorders. The most prevalent fine-level disorders of WHWTs were periodontal disease, otitis externa, overgrown nails, allergic skin disorder and obesity. At a grouped level, the most common disorders were cutaneous, dental, aural, claw/nail and musculoskeletal. These results reiterate the power of primary-care records to highlight common events within breeds and expand the evidence-base on breed-related health in dogs [18]. The findings can provide breeders, veterinary practitioners and owners with a generalisable evidence-base to improve health and welfare for WHWTs.

The WHWT showed a median longevity of 13.4 years in the current study, which is in line with a previous report of 13.5 years for the WHWT and higher than the median longevity of 12.0 years reported across all breeds [3]. As a smaller-sized breed, the longevity of the WHWT benefits from the inverse association between increasing bodyweight and longevity [3, 28]. It is worth noting that longevity values using the methodology of the current study will be biased upwards for breeds such as the WHWT that are declining in popularity whereas the converse effect will occur for breeds that are rising in popularity [29]. The median longevity of males (13.8 years) was almost 1 year longer than females (12.9 years). This male longevity advantage in the WHWT is unusual across dog breeds and is in contrast to previous reports in individual breeds that reported no sex difference in Labrador Retrievers [25] or French Bulldogs [30] or that conversely showed a female advantage for Rottweilers [27] and German Shepherd Dogs [26]. These contrasting results across breeds demonstrates that the female longevity advantage that has been previously reported in dogs is not universal across breeds and each breed needs to be considered individually in terms of sex-associated longevity [31].

It is important to distinguish between prevalence and predisposition when interpreting results from epidemiological studies [6]. Prevalence is an absolute value that defines the overall frequency of a condition whereas predisposition is a relative value that describes the risk in one group compared to another [32]. A disorder may be highly prevalent within a particular breed (i.e. the disorder is common in the breed) and therefore can be considered as an important issue for the welfare of the breed without necessarily exhibiting a specific predisposition (i.e. the disorder is no more common in this breed than in dogs overall). Conversely, the breed may be predisposed to the disorder (i.e. the breed has a much higher relative risk of the disorder than dogs overall) but without the disorder being common (i.e. it need not be important to the breed) [33]. For the purposes of the current paper, an increased probability of disease compared with dogs overall or with breeds of a similar body size was accepted as evidence of disease predisposition [6] and preference was given to discussion of disorders that were either or both common and predisposed.

Lower respiratory tract disease and neoplasia were the most common causes of death in WHWTs in the current study, each accounting for 10.2% of deaths. Given that neoplasia accounted for 16.5% of deaths in dogs overall and 14.7% of deaths in Miniature Schnauzers, a similar-sized breed to the WHWT, in previous VetCompass reports [3, 33], the current study does not suggest that the WHWT is predisposed to neoplasia in general. The prevalence of lower respiratory tract disease, however, was much higher in the current study than a previous report estimating mortality due to respiratory conditions at 3.9% across all breeds [3]. This suggests that lower respiratory disease is a significant life-ending issue for the WHWT and this view is supported by the literature. An owner survey in the US reported that 10.5% of WHWTs died with pulmonary fibrosis [5]. Idiopathic pulmonary fibrosis (IPF) is a chronic, progressive interstitial lung disease that carries a poor prognosis and the WHWT has been reported as strongly predisposed [34, 35]. Therefore, it is possible that IPF accounted for much of the mortality due to lower respiratory tract disease seen in the current study.

The most prevalent fine-level disorder in the current study was periodontal disease, with 15.7% of WHWTs affected. This is comparable to results for the Cavalier King Charles Spaniel (15.2%) and Border Terrier (17.6%) but higher than values reported for larger sized breeds such as Labrador Retriever (4.2%) [25], German Shepherd Dog (4.1%) [26] and Rottweiler (3.1%) [27]. The prevalence of periodontal disease has been reported to increase with age, decrease with increasing body size [36] and increase with dental malocclusion [37]**.** The KC breed standard describes the WHWT with teeth “large for size of dog” [1] which could predispose to malocclusion and therefore promote dental disease. Indeed, misplaced lower canines are cited in the KC Breed Watch system as points of concern for special attention by judges [9]. Consequently, the ageing population in the current study, the smaller size of breed and the dental profile of WHWTs contribute to make the 15.7% prevalence of periodontal disease unremarkable and suggests that dental disease is a common and important disease for the WHWT but that the breed is not particularly predisposed to the condition compared with other similar sized breeds.

Six of the 27 most common fine-level disorders (otitis externa, allergic skin disorder, pododermatitis, pyoderma, atopic dermatitis and dermatitis) represent various clinical manifestations of inflammatory skin disease, and have a combined prevalence of 28%. Whilst otitis externa, pododermatitis and pyoderma may occur in isolation and may be aetiologically unrelated, they frequently represent manifestations or complications of an underlying allergic skin disease, specifically atopic dermatitis [38, 39]. The terms allergic skin disease, atopic dermatitis and dermatitis represent differing levels of aetiological specificity [38]. If these six common fine-level disorders are thus viewed as related to each other within the context of allergic, and more specifically atopic, dermatitis, their combined prevalence in this study may conceal a higher prevalence of true atopic dermatitis in the UK population than the 2.5% prevalence of formally diagnosed atopic dermatitis reported in this study might suggest. Indeed, based on a systematic review of frequency data from different populations, the WHWT has been identified as globally predisposed to atopic dermatitis [10], and 23.2% of WHWTs in the US were described as affected in an owner reported survey [5]. Methodological differences between studies reporting disease frequency data render direct comparisons difficult [10].

Diagnostic term substitution can describe the phenomenon whereby multiple correct but varying alternative biomedical diagnostic terms may be recorded appropriately for any single disorder event. As described above, this phenomenon may result in apparent underrepresentation of a complicated disease such as atopic dermatitis in this study when many true instances of the disease may be recorded using other terms that fall under the diagnostic umbrella relating to allergic dermatitis. This phenomenon may also affect breeds differentially, as breed-specific phenotypes of atopic dermatitis are recognized [11, 39]. WHWTs are reportedly more often affected with widespread skin lesions than other atopic dogs [39], and as a result may show underreporting of associated distinct disorders such as otitis externa. Otitis externa was the second most prevalent disorder diagnosed in WHWT, affecting 10.6% of the current study population which is comparable to the 10.2% prevalence reported in dogs overall [23]. A survey of owners conducted by the KC reported a much lower prevalence of 2.87% in the WHWT [4] although biases in owner reporting may in part account for this difference [40]. In summary, the complexity of allergic skin disease in terms of its aetiologies, diagnostic definitions, criteria and associated skin infections [12, 38, 41, 42], in combination with the variable use of terminology in clinical practice, add to the challenge of accurately documenting the frequency of this group of disorders from primary care clinical records.

It is noteworthy that the prevalence of cutaneous disorders at a grouped level was 22.7%, which is higher than the 15.5% reported across all dogs [23] and also higher than previous reports on other similarly small-sized breeds including French Bulldog (17.9%) [30], Pug (15.6%) [43] and Border Terriers (10.2%) [44]. Therefore, this study supports the body of evidence that skin disorders are a significant health issue for the WHWT, albeit that the current study does not highlight the predisposition to be as marked as previously reported. This difference may relate to internationally differing disease profiles in breeds because many of the previous studies were conducted outside the UK. It is also possible that the decreasing popularity of the WHWT over the past decade may have reduced the selection pressure on the breed and allowed the use of genetically and physically healthier stock such that the dermatological health of the breed is genuinely improving.

Other previously reported breed predispositions in the WHWT include keratoconjunctivitis sicca (KCS), cranial cruciate ligament disease and diabetes mellitus [45–47]. In the current study, the prevalence of KCS was 2.0%, cruciate disease was 1.8% and diabetes mellitus was 1.2%. Although these conditions were not at the top of the list of the most common disorders within the breed, each of these results support a breed predisposition compared with prevalence values in other small sized breeds [30, 33, 43, 44]. The results also suggest that these diseases are important clinically to the WHWT because of their reasonably high absolute prevalence values. For these reasons, these disorders warrant inclusion within the lists of priority disorders for control and management in the WHWT by breeders, veterinarians and owners.

Some sex-related differences were identified in the WHWT in the current study. Male WHWTs were more likely than females to be diagnosed with otitis externa (12.2% vs 8.7% respectively) and aggression (2.8% vs 1.1% respectively). A sex-related difference in otitis externa has not been previously identified in breed studies that used a similar design to the current study [25–27, 33]. However, a male predisposition to aggression is supported by a substantial body of evidence [26, 27, 48, 49]. Conversely, females had a higher prevalence of periodontal disease than males (17.7% vs 14.0% respectively). A female predisposition to periodontal disease was also identified in the Miniature Schnauzer [33]. The discovery and reporting of sex-based prevalence differences highlight that certain disorders may benefit from specific focus on preventive and remedial control within sexes to optimise health and welfare improvements. No sex association was detected for atopic dermatitis, which is in agreement with a previous study exploring risk factors for the disease [50].

The ageing population in the current study, with a median age of 7.8 years for the WHWTs overall, reflects declining popularity of the breed in the wider general population of dogs. A similar pattern of declining KC registrations in recent years has been reported [2]. It is possible that the veterinary profession may have contributed to declining popularity of WHWTs by emphasising poor health in the breed to their clients, especially in relation to skin disease. However, the frequency of skin disease in the current study is not as marked as might be expected. It is possible that veterinary opinion has been influenced by an element of cognitive bias because veterinary clinicians are presented with the most severely affected and recurrent cases. The results of the current study might suggest that the decline in popularity of the breed has led to more regulated breeding and thus a reduction in inherited cutaneous disorders. This could be an area for future research and is relevant to breeds such as the Pug, French Bulldog and Bulldog, which are currently increasing in popularity, but may face declining populations in the future for similar health-associated reasons [30, 43].

The application of “big data” using anonymized clinical records from primary-care veterinary practice is radically changing how epidemiological research on companion animals is conducted [19, 51–53]. Previously, much of the data used in companion animal research were sourced from referral practice, pet insurance databases or from questionnaires that are subject to substantial selection bias. Although useful, studies based on these resources often had limited scope to generalize results from these skewed populations under examination to the wider general population [18]. Although also not without limitations, primary-care data research offers much better generalizability. To date, primary-care clinical data on companion animals have offered opportunities for research on prevalence in species overall [23, 54] as well as within individual breeds [33]. These data have also been used for studies to explore specific disorders [55, 56], drug therapy [57], longevity [3], demography [58], methodology [18, 59–61] and human translational studies [62]. The current study adds to this increasing body of evidence derived from primary care clinical data that is providing a new perspective on the health and care of companion animals.

There were limitations to methodology in the current study, some of which have been explored previously [33, 63]. A final biomedical diagnosis is not always reached, or often even required, in primary-care veterinary practice for successful clinical management [64, 65]. Consequently, many ‘diagnoses’ reported in the current study were, in reality, presenting signs. As discussed above, fragmentation of disorders recorded across multiple terms may have resulted in under-estimation of some precise diagnostic terms such as atopic dermatitis. Analysis and reporting at the precision of both fine-level and grouped-level terms aimed to limit the inferential impact of this diagnostic hierarchical phenomenon [23]. There are a proportion of dogs in the general population that are not registered with, or likely to present to, primary-care veterinary practices. If the prevalence of, and risk factors for, disease in this group differs to the subset that are under veterinary care, then the results of the current study may not generalise well to this unrecorded group. As discussed, the median age of WHWTs in the current study was 7.8 years and therefore the results may be skewed towards disorders of older dogs.

## Conclusion

This study of over 6,000 WHWTs under primary veterinary care highlighted that, despite a recent decline in popularity, the breed is still relatively common in the UK. The WHWT is a long-lived breed. Lower respiratory tract disease is a common cause of death and therefore warrants consideration as a significant health issue within the breed. The most prevalent disorders identified were periodontal disease, otitis externa, overgrown nails, allergic skin disorder and obesity. Some important sex-associated differences were identified, with males living significantly longer than females. Although cutaneous disorders were the most common disorder in the breed at a grouped level, their prevalence was lower than might be expected which may suggest that the negative impact of cutaneous disease on the breed is waning. The study reiterates the power of primary-care veterinary clinical records for research to help understand breed health in dogs and to support evidence based approaches towards improved health and welfare in dogs.

## Data Availability

The datasets generated during and/or analysed during the current study will be made available at the RVC Research Online repository.

## Abbreviations

CI: Confidence interval
EPR: Electronic patient record
IPF: Idiopathic pulmonary fibrosis
IQR: Interquartile range
KC: The Kennel Club
KCS: Keratoconjunctivitis sicca
OR: Odds ratio
