# Correction to: West Highland White Terriers under primary veterinary care in the UK in 2016: demography, mortality and disorders

**DOI:** 10.1186/s40575-019-0077-0

**Published:** 2019-10-28

**Authors:** Dan G. O’Neill, Zoie F. Ballantyne, Anke Hendricks, David B. Church, Dave C. Brodbelt, Camilla Pegram

**Affiliations:** 1 Pathobiology and Population Science, The Royal Veterinary College, Hawkshead Lane, North Mymms, Hatfield, Herts AL9 7TA UK; 2 Clinical Sciences and Services, The Royal Veterinary College, Hawkshead Lane, North Mymms, Hatfield, Herts AL9 7TA UK

**Correction to: Canine Genet Epidemiol (2019) 6:7**

**https://doi.org/10.1186/s40575-019-0075-2**

In the original publication of this article [1], due to an error in a single count relating to the denominator used for this study, some of the derived values were wrong, so that abstract, plain English summary, results and Fig. 1 all need to be revised.

**Abstract**

Update from

Results: WHWTs comprised 6605/905,544 (0.7%) dogs under veterinary care during 2016 from 886 clinics.

to

Results: WHWTs comprised 6605/336,865 (1.96%) dogs under veterinary care during 2016 from 438 clinics.

**Plain English Summary**

Update from

WHWTs comprised 6605 (0.7%) of the overall 905,544 study dogs.

to

WHWTs comprised 6605 (1.96%) of the overall 336,865 study dogs.

**Results**

Demography and mortality

Update from

The study population of 905,544 dogs under veterinary care attending 886 clinics in the vetCompass database during 2016 included 6605 (0.7%) WHWTs. Annual proportional birth rates showed that WHWTs decreased in popularity from 1.69% of the annual VetCompass birth cohort in 2004 to 0.43% in 2015 (Fig. 1).

to

The study population of 336,865 dogs under veterinary care during 2016 at 438 clinics in the VetCompass database included 6605 (1.96%) WHWTs. Annual proportional birth rates showed that WHWTs decreased in popularity from 4.79% of the annual VetCompass birth cohort in 2004 to 0.90% in 2015 (Fig. 1).

**Figure 1 Legend**

Update from

Fig. 1 Annual proportional birth rates (2004–2015) for West Highland White Terriers (*n* = 6605) among all dogs (*n* = 905,544) under UK primary veterinary care from January 1st 2016 to December 31st, 2016 at practices participating in the VetCompass™ Programme

to

Fig. 1 Annual proportional birth rates (2004–2015) for West Highland White Terriers (n = 6605) among all dogs (*n* = 336,865) under UK primary veterinary care from January 1st 2016 to December 31st, 2016 at practices participating in the VetCompass™ Programme

The authors apologize for any confusion this may have caused.
